# Exosomes from Adipose-Derived Stem Cells (ADSCs) Overexpressing miR-21 Promote Vascularization of Endothelial Cells

**DOI:** 10.1038/s41598-019-49339-y

**Published:** 2019-09-06

**Authors:** Yang An, Jianfang Zhao, Fangfei Nie, Zelian Qin, Hongyu Xue, Guanhuier Wang, Dong Li

**Affiliations:** Department of Plastic Surgery, Peking University Third Hospital, No. 49 Huayuan North Road, Haidian District, Beijing, 100191 China

**Keywords:** Genetics research, Stem-cell research

## Abstract

In the past few years, exosomes released from adipose-derived stem cells (abbreviated as ADSCs) have shown promises to provide therapeutic benefits in the fields of regenerative medicine. miRNAs, existing in exosomes, are endogenous, small noncoding RNAs that play important roles in a variety of cellular functions and tumor development. Emerging evidences have indicated that miR-21 is one of the important miRNAs associated with tumor angiogenesis. In this study, we identified the role of exosomes from ADSCs overexpressing miR-21 in regulating/promoting vascularization of endothelial cells. Experimental data indicated an elevated miR-21 level in exosomes released by ADSCs overexpressing miR-21. *In vitro* matrigel angiogenesis assay showed that exosomes secreted by ADSCs overexpressing miR-21 significantly promoted the vascularization of HUVEC cells (an endothelial cell line). Quantitative real-time polymerase chain reaction (qRT-PCR) and western blot (WB) revealed an upregulation of HIF-1α, VEGF, SDF-1, p-Akt, p-ERK1/2 and downregulation of PTEN in response to miR-21 overexpression, indicating that miR-21 enriched exosomes induced angiogenesis through Akt and ERK activation and also HIF-1α and SDF-1 expression. Our work suggests that exosomes from ADSCs that overexpressing miR-21 can potentially promote vascularization and therefore the transplantation of exosomes from their culture may be suitable for clinical effort in regenerative medicine.

## Introduction

Exosomes are microparticles with a diameter 30–100 nm that are released by cells and contain proteins, lipids, and RNAs (mRNA and miRNA)^1–3^. The effect of exosomes from different cells varies significantly because the functional components vary depending on their cell of origin^4,5^. For example, exosomes derived from bone marrow dendritic cells with tumor associated peptides exhibit antitumor activity *in vivo* by attracting T cells in an antigen-specific manner and stimulating the antitumor activity of cytotoxic lymphocytes^6^. Exosomes secreted by macrophages are able to promote breast cancer invasion and metastasis^7^. Exosomes can also promote or inhibit angiogenesis depending on their cell of origin^8^—activated T lymphocyte exosomes promote angiogenesis^9^ while those from apoptotic T cells inhibit angiogenesis^10^. Therefore, exosomes from different cells can be used or combined with drugs for different therapeutic purposes^11–13^.

Adipose-derived stem cells (ADSCs), a class of multipotent cells, can be readily isolated and cultured in large quantity from subcutaneous adipose tissue using well-established protocols^14–18^. There have been numerous successes in using ADSCs for tissue engineering^19–21^. For example, ADSCs can be used for increasing survival of fat grafts or soft tissue flaps^22^, or regenerating skeletal muscles^23^. While many people have sought to use stem cells as a promising way to heal human tissue, exosomes from ADSCs show more promises to provide therapeutical benefits: (i) due to the physiochemical stability in the body and their multidimensional packaging, exosomes make great models for therapeutic medicine^24^; (ii) exosomes represent a class of cell-free regenerative medicine, which is safer because stem cells sometimes pose a serious safety risk owing to potential tumorigenicity^24^; (iii) exosomes can be readily produced in large quantity in laboratory setting with well-established protocols^25^; (iv) the specificity of the exosome components endows them a cell-specific manner^26^; (v) the loaded miRNA, proteins and other component in exosomes are less prone to cellular degradation^27^, and (vi) the functions of exosomes are easy to be tailored by making modification of the cells of origin. In this work, we intend to use ADSC-derived exosomes to promoting vascularization for regenerative purpose.

MicroRNAs (miRNAs), regulating gene expression at the post-transcriptional level, can affect a variety of physiological functions, including development^28^, cell differentiation^29^, proliferation^30^, and apoptosis^31^. As a result, miRNAs can also regulate the genesis and development of cancer by functioning as a kind of tumor suppressor gene or oncogene^32^. miR-21 is one of such genes, found to have a high expression level in many solid tumors^33^. Emerging evidences suggest that miR-21, known as an ongcomiR, exerts their effects at multiple steps in the metastatic cascade by influencing cancer cell adherence^34^, migration^35^, invasion^36^, and motility^37^. Recent evidences suggest a role of miR-21 in tumor angiogenesis^38–42^.

**Figure 1:**
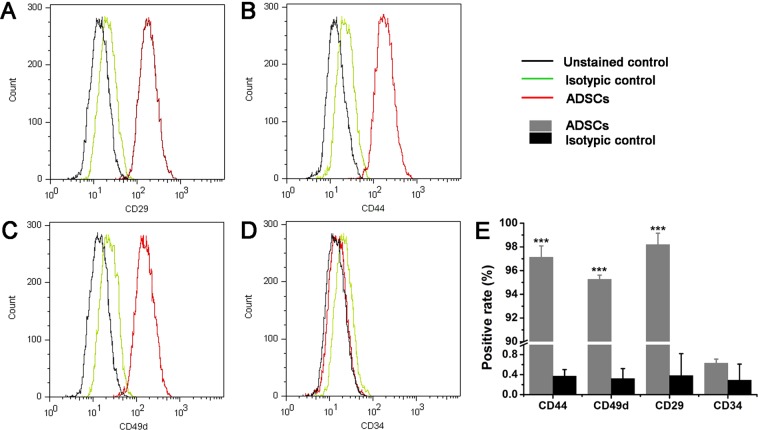
Characterization of ADSCs isolated from inguinal fat pad of 3-week old Lewis male rats by flow cytometric analysis. (**A**–**D**) Flow cytometric analysis of ADSC surface markers (CD29, CD44, CD49d and CD34). The results presented are typical of those obtained from three separate experiments. (**E**) Summary of the result in (**A**–**D**). Error bars were calculated based on triplicates. (^***^means P < 0.001 vs. isotypic control).

A number of studies have indicated that various stem cells release exosomes that exert functional effects that mimic the effect of their parental cells of origin^43,44^. In this work, we, thus, evaluated the therapeutic effect of exosomes secreted by ADSCs overexpressing miR-21 on regulating vascularization of endothelial cells for regenerative and therapeutic goal (as shown in Fig. 1A). *In vitro* matrigel angiogenesis assay showed that exosomes secreted by ADSCs isolated from inguinal fat pad of 3-week old Lewis male rats and overexpressing miR-21 significantly promoted the vascularization of HUVEC cells. Mechanistic study indicated that miR-21 overexpressed exosomes induce angiogenesis through Akt and ERK activation and also HIF-1α and SDF-1 expression. Our work suggests that exosomes from ADSCs that overexpressing miR-21 can potentially promote vascularization and therefore the transplantation of exosomes from their culture may be suitable for clinical effort in regenerative medicine.

## Result and Discussion

### Isolation and identification of ADSCs

Following the criteria for identifying ADSCs^45^, we measured the relative cell-surface abundance of each biomarker by flow cytometry using commercial antibodies. Gratifyingly, analysis of surface antigen expression demonstrated that the isolated ADSCs at 4^th^ passages were highly positive for mesenchymal stem cell surface markers CD29 and CD44, stromal cell surface maker CD49d, but negative for hematopoietic stem cell surface marker CD34 (Fig. 1 and Supplementary Figs S1–S4), indicating that ADSCs were successfully isolated and ready for assays.

### Isolation and identification of exosomes

**Figure 2:**
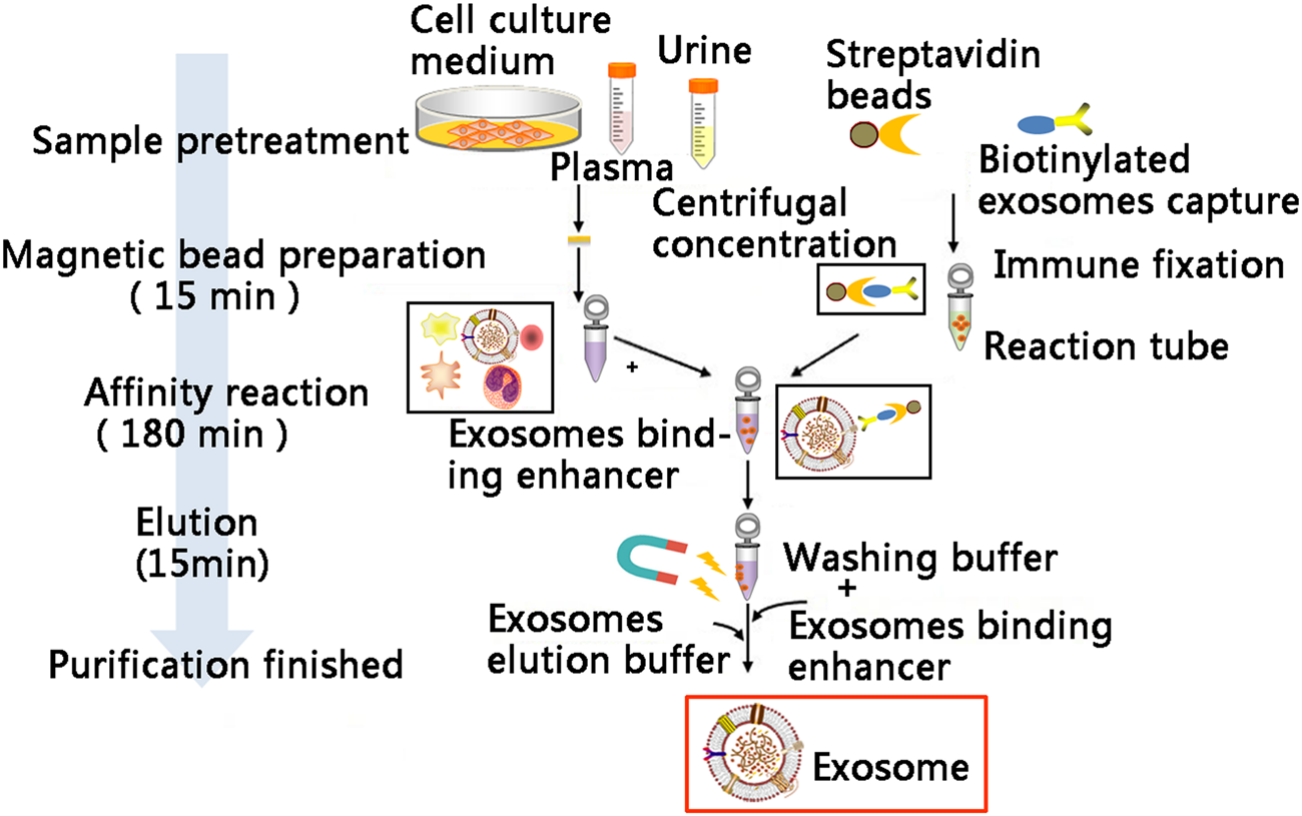
The protocol for exosome extraction from ADSCs.

**Figure 3:**
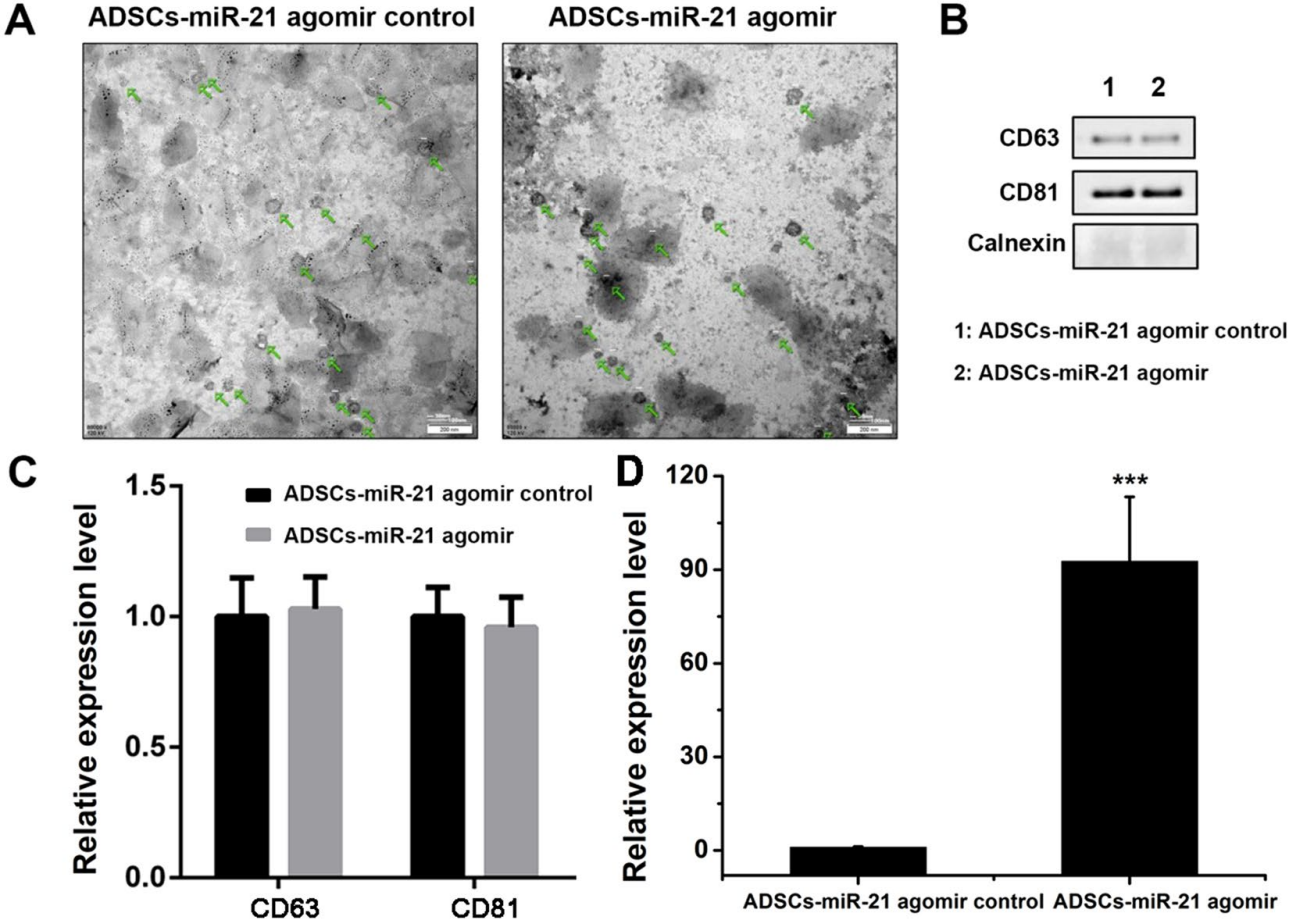
Characterization of exosomes derived from ADSCs isolated from inguinal fat pad of 3-week old Lewis male rats by flow cytometric analysis. (**A**) TEM images show the ultrastructure of ADSC-derived exosomes. Scale bar was 200 nm. (**B**) Expression of the exosome markers CD63, CD81, and Calnexin confirmed by western blot. (**C**) Quantification of the expression of the exosome markers CD63 and CD81 in (**B**). (**D**) The level of miR-21 in exosomes secreted from ADSCs with or without miR-21 overexpression. Error bars were calculated based on triplicates. (^***^means p < 0.001 vs. ADSCs- miR-21 agomir control).

Exosomes from ADSCs were purified as shown in Fig. 2. We then used transmission electron microscope (TEM) to identify the morphology of exosomes extracted from ADSCs culture. As shown in Fig. 3A, exosomes (indicated by green arrow) released by control ADSCs (ADSCs-miR-21 agomir control) or those overexpressing miR-21 (ADSCs-miR-21 agomir) are both hollow spherical microvesicles with a diameter of 30–100 nm. Obviously, there is no difference in numbers of those secreted exosomes either. Using BCA protein quantification assay, we confirmed that there’s no obvious difference in total protein amount (282.2 v.s. 289.2 µg) in the exosomes isolated from control and miR-21 overexpressing ADSCs (Supplementary Fig. S5). Western blot reveals the presence of CD63 and CD81, two exosomal markers and the absence of calnexin, a negative control for an exosome protein marker (Fig. 3B,C). qPCR confirmed a significant enrichment of miR-21 in ADSCs-miR-21 agomir-exosomes (Fig. 3D, Supplementary Fig. S6). All these data indicate that exosomes were successfully isolated.

### Exosomes from ADSCs overexpressing miR-21 promote vascularization of endothelial cells HUVEC

**Figure 4:**
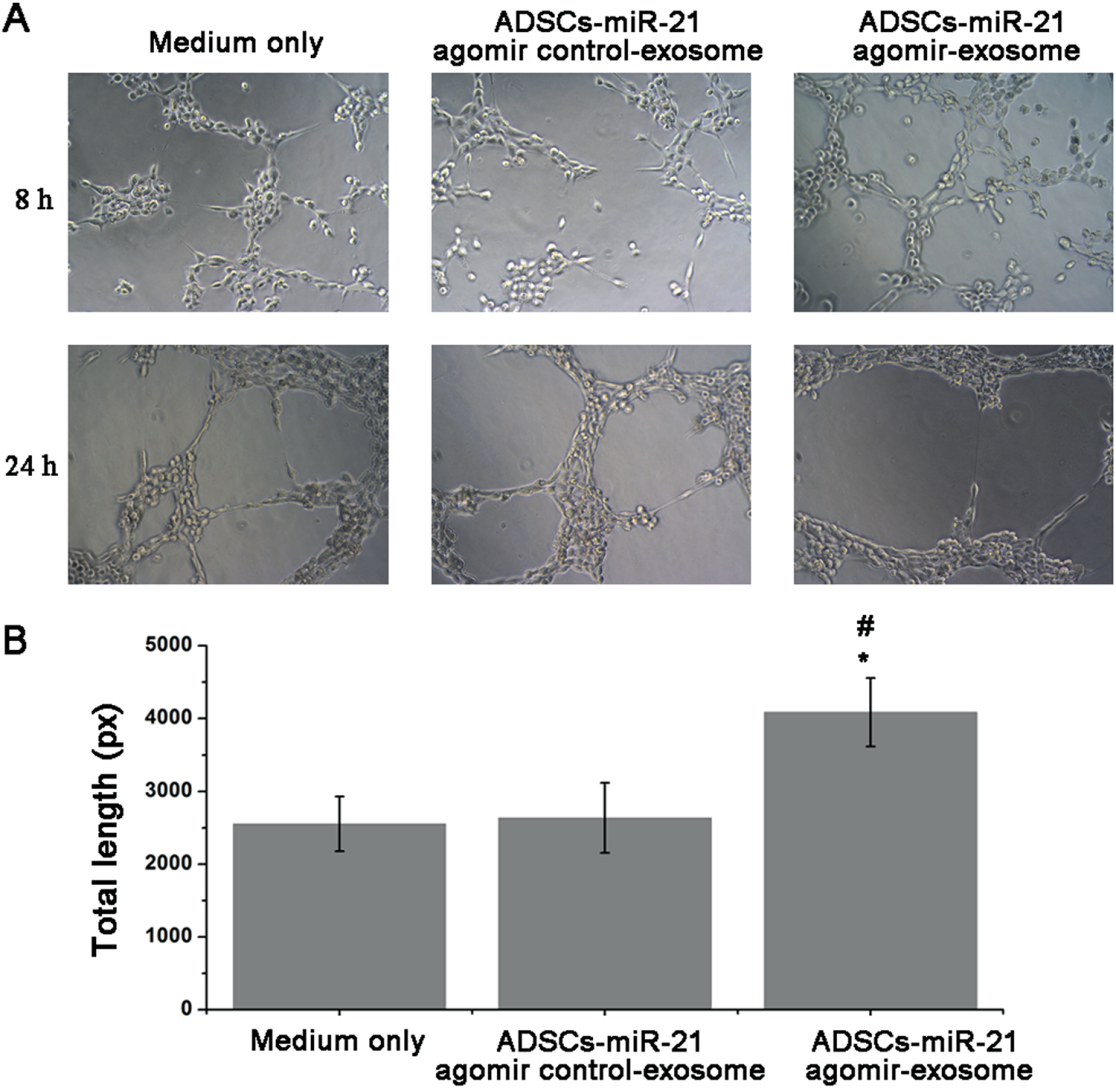
Exosomes from ADSCs overexpressing miR-21 promote vascularization of endothelial cells (HUVEC). (**A**) Tube formation capability detected in endothelial cells (HUVEC) stimulated with 5 μg/ml ADSCs-miR-21 agomir control-exosome or ADSCs-miR-21 agomir exosome for 8 or 24 h. (**B**) Quantification of the length of the formed tubes at 8 h in (**A**). Error bars were calculated based on triplicates. (^*^means P < 0.05 vs. medium only; ^#^means P < 0.05 vs. ADSCs- miR-21 agomir control-exosome).

**Figure 5:**
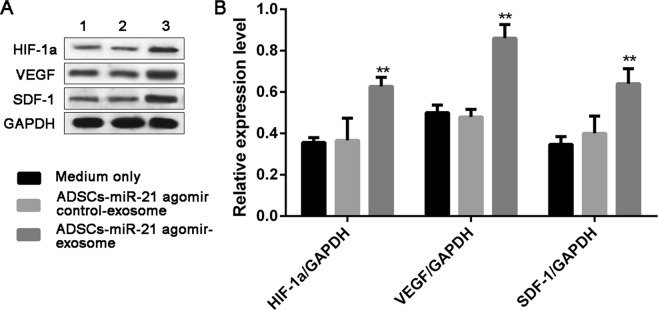
Exosomes from ADSCs overexpressing miR-21 enhanced expression of HIF-1α, VEGF and SDF-1 in HUVEC. Western blot (**A**) and its quantification (**B**) of the expression of HIF-1α, VEGF, SDF-1 in HUVEC with control medium (**1**), exosomes from control ADSCs (**2**) and exosomes from miR-21 overexpressing ADSCs. Error bars were calculated based on triplicates. (^**^Means p < 0.01 vs. both medium only and ADSCs- miR-21 agomir control-exosome).

We then tested the tube formation of endothelial cells HUVEC treated by exosomes isolated from ADSCs-miR-21 agomir control and ADSCs-miR-21 agomir. Compared with control group without exosome treatment (no tube formation), HUVEC cells treated by exosomes overexpressing miR-21 showed more tube formation than those cells treated by ADSCs- miR-21 agomir control-exosomes at 8 h (Fig. 4A). And after 24 hours, no obvious difference was observed in the HUVEC culture treated by ADSCs- miR-21 agomir control-exosomes while HUVEC cells treated by ADSCs- miR-21 agomir showed largest meshes. The length of tubes formed can be easily quantified as a measure of *in vitro* vascularization. As shown in Fig. 4B, the total length of tubes formed by HUVEC cells treated by ADSCs-miR-21 agomir is significantly longer than the control groups (also see in Supplementary Table S1). Furthermore, we did the expression analysis of HIF1-α, VEGF and SDF-1 in HUVEC after being treated by ADSCs-miR-21 agomir control exosome or ADSCs-miR-21 agomir exosome for 24 h. The results in Fig. 5 indicated that the expression of these three proteins are enhanced by ADSCs-miR-21 agomir exosome treatment (1.68-fold, 1.79-fold and 1.6-fold for HIF1-α, VEGF and SDF-1, respectively). All these results indicate that exosomes isolated from ADSCs overexpressing miR-21 can promote the vascularization of endothelial HUVEC cells.

### Overexpression of miR-21 in ADSCs leads to the upregulation of HIF1-α, VEGF, SDF-1, p-Akt, p-ERK1/2 and downregulation of PTEN

**Figure 6:**
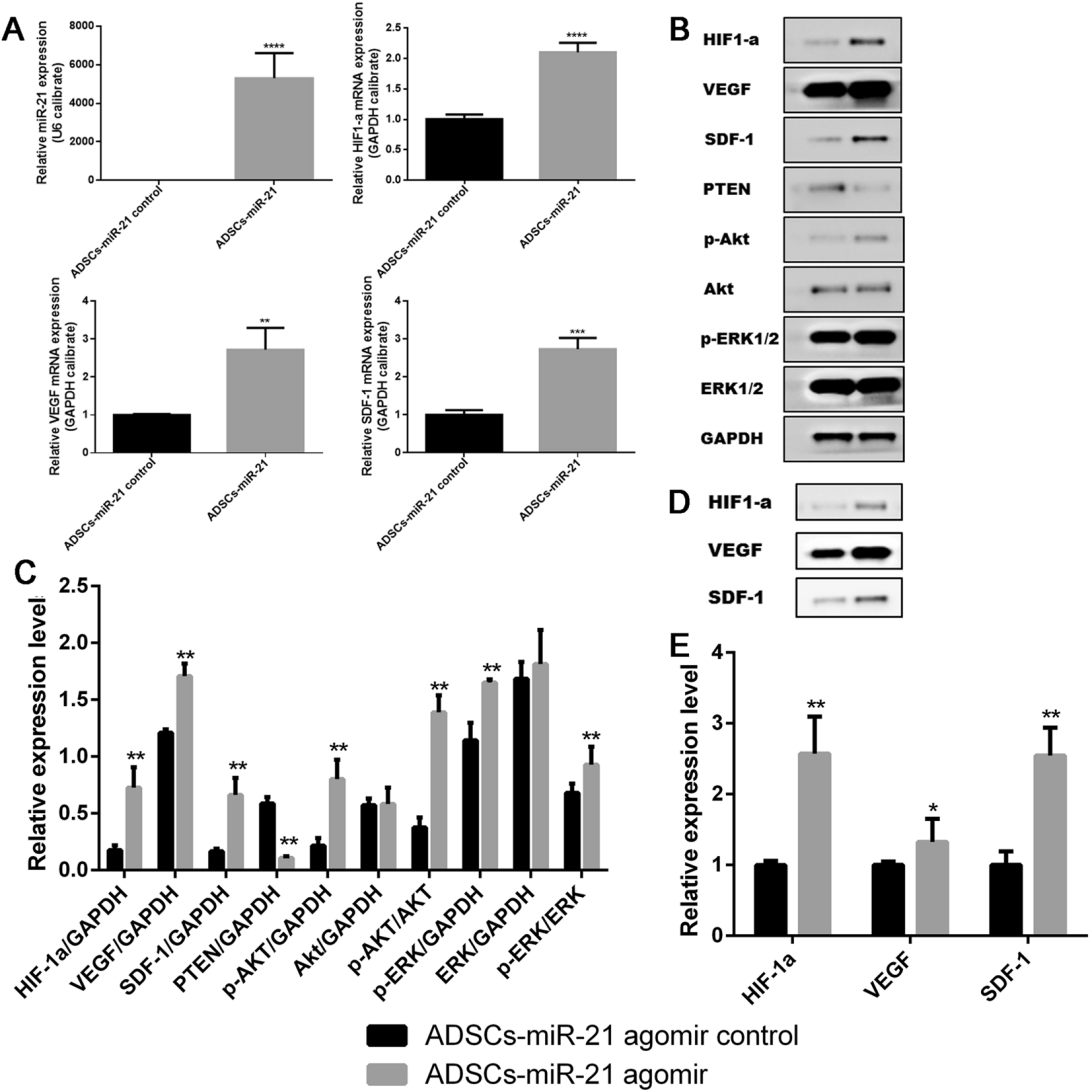
Overexpression of miR-21 in ADSCs leads to the upregulation of HIF-1α, VEGF, SDF-1, p-Akt, p-ERK1/2 and downregulation of PTEN. (**A**) Quantification of the expression level of miR-21, HIF-1α, VEGF, SDF-1 in ADSCs with and without miR-21 overexpression. (**B**,**C**) Western blot and its quantification of the expression level of HIF-1α, VEGF, SDF-1, PTEN, p-Akt, Akt, p-ERK1/2, ERK1/2 in ADSCs with (**2**) and without miR-21 overexpression (**1**). (**D**,**E**) Western blot and its quantification of the expression level of HIF-1α, VEGF, SDF-1 in exosomes isolated from ADSCs with (**2**) and without (**1**) miR-21 overexpression. Error bars were calculated based on triplicates. (^***^means p < 0.001 vs. ADSCs- miR-21 control, ^**^means p < 0.01 vs. ADSCs- miR-21 control, ^*^means p < 0.05 vs. ADSCs- miR-21 control).

To investigate the potential mechanism that exosomes from ADSCs overexpressing miR-21 promote vascularization, we used qRT-PCR to quantitatively characterize some relevant targets on ADSC cells, including HIF-1α, VEGF, SDF-1 (Supplementary Fig. S7). In response to hypoxia, stimulation of growth factors, and activation of oncogenes as well as carcinogens, HIF-1α is overexpressed and/or activated and targets those genes which are required for angiogenesis, metabolic adaptation to low oxygen and promotes survival^46^. HIF-1α has also been taken as a key factor in regulation of VEGF, an angiogenic factor^47^. In addition, SDF-1 is reported to induce angiogenic activity^48^. As shown in Fig. 6A, all these three genes are significantly upregulated in response to the overexpression of miR-21, indicating miR-21 promotes angiogenesis by regulating HIF-1α-involved cell signaling and SDF-1 expression.

We also checked the variation of Akt, PTEN, ERK1/2 in response to the overexpression of miR-21 on ADSCs by western blot, in addition to HIF-1α, VEGF, SDF-1. According to western blot (Fig. 6B,C),HIF-1α, VEGF, SDF-1 are significantly upregulated, which is consistent with qRT-PCR result. In addition, there was an obvious increase in p-Akt expression, moderate increase in p-ERK1/2 expression while the amount of Akt and ERK1/2 didn’t change much. Meanwhile, the expression level of PTEN significantly decreased. We observed similar protein variation trend (i.e., upregulation of HIF-1α, VEGF and SDF-1) in exosomes derived from ADSCs overexpressing miR-21(Fig. 6D,E). Altogether, these results indicate that overexpression of miR-21 leads to HIF-1α overexpression in combination with downregulation of tumor suppressor genes such as PTEN, and amplification of oncogenes (Akt, and ERK1/2) to promote angiogenesis. In addition, miR-21 enriched exosomes induced angiogenesis also through SDF-1 expression.

## Conclusion

**Figure 7:**
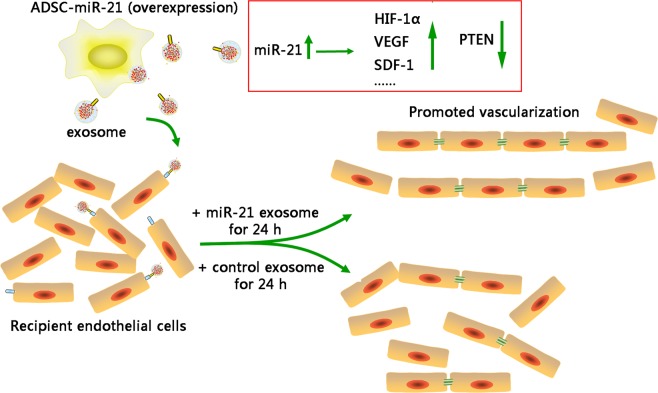
The graphic summary of miR-21 overexpressing exosome promoting vascularization of endothelial cells.

miR-21 has been indicated to be an important regulator of angiogenesis, involving in regulating the proliferation and migration of vascular cells, like endothelial cells. Direct evidences have shown that downregulation of miR-21 expression significantly reduces the proliferation and migration of HUVECs, and conversely, miR-21 overexpression significantly enhances HUVECs proliferation and migration, indicating the importance of miR-21 on angiogenesis. In spite that miR-21, as a circulating tumor biomarker, exerts its effect at multiple steps in cancer metastasis by affecting adhesion, migration, invasion, and angiogenesis, we are still able to make the most utilization of it for therapeutic purpose by integrating its function with ADSC exosomes. Here we used exosomes from ADSCs in combination with miR-21 overexpression to promote the vascularization of endothelial cells for regenerative purpose and demonstrated that miR-21 induces tumor angiogenesis through targeting PTEN, leading to activate AKT and ERK1/2 signaling pathways, and thereby enhancing HIF-1α and VEGF expression (Fig. 7). Our results demonstrate that exosomes secreted by ADSCs overexpressing miR-21 could possibly be used to assist wound healing by improving vascularization. Exosome therapy in combination with onco-miRs may represents a multi-faceted, paradigm-shift strategy for promoting regenerative tissue engineering and carry huge expectations for medical and clinical application in the near future.

## Materials and Methods

### Reagents

Monoclonal antibodies against CD29 (HMb-1, FITC) was obtained from eBioscience (#11-0291-80), CD44 Monoclonal Antibody, OX-50, FITC and CD34 Polyclonal Antibody were purchased from Invitrogen (MA5-16906 and PA5-47849). Akt was obtained from CST (#4685), CD49D from Miltenyi (#130-111-487), CD63, Calnexin, CD81 from SANTA (SC-15363, SC-70481, SC7637). Protease inhibitor cocktail was obtained from Shanghai Yuanye Bio-Technology Co., Ltd (# 10557), DEPC from Amresco (E174), DMEM/F12 from Gibco (# 12400-024), ECL from Thermo (NCI5079), ECM from Science (#1001), FBS from Gibco (#16000-044), GAPDH from abclonal (AC002), Hc1 from Xinyang Chemical Reagent Factory (GB622-89), HIF1-α from SANTA (sc-71247), HUVEC from Chinese Academy of Medical Sciences Basics Medical Science Institute. Other reagents include Matrigel Basement Membrane Matrix (BD, #356234), p-Akt (SCT, #4060), p-ERK1/2 (SANTA, CS-81492), PMSF (Amresco, #329-98-6), PTEN (CST, #9559), PVDF membrane (Millipore, IPVH00010), SDF-1 (SANTA, SC6193), SYBR green qPCR assay kit (Thermo, K0221), TEMED (Amresco, #00761), TRI Reagent BD (MRCgene, TB0126), VEGF (Affinity, AF5131).

### Cell isolation and culture

ADSCs were isolated from inguinal fat pad of 3-week old Lewis male rats as previous description^49^. All animal experiments in this work were performed in accordance with the guidelines of the Administration of Experimental Animals (Beijing, revised Dec. 2004) and approved by the Animal Ethics Committee of Peking University Third Hospital. Once isolated, ADSCs were maintained in the complete growth medium (Dulbecco’s Modified Eagle’s Medium (DMEM, Invitrogen) containing L-glutamine, supplemented with 10% fetal bovine serum (FBS), 1% penicillin/streptomycin (Invitrogen)), at 37 C in a fully humidified incubator with 5% CO_2_. The initial cell density was 1.5 × 10^4^ cells/cm^2^ and medium was changed after the first 24 hours. ADSCs were subcultured/split at 100% confluency and otherwise medium was changed every 2–3 days. ADSCs at 3^rd^ to 6^th^ passages were used for assays mentioned in this manuscript.

### Cell characterization

At 80–90% confluency, ADSCs were lifted with trypsin and filtered with a cell strainer with 100 µm pores. The cells were then spinned down at 2000 rpm for 5 min and resuspended in pre-chilled PBS buffer at 2 × 10^5^ cells/mL density. Cells were stained with Armenian hamster anti-CD29 (1:500), mouse anti-CD44 (1:500), mouse anti-CD49d antibody (1:500), sheep anti-CD34 antibodies respectively at 4 C overnight, before being washed by pre-chilled PBS twice. ADSCs were stained with FITC labeled secondary antibodies at room temperature for 30 min and washed by PBS buffer twice in prior to applying on flowcytometry. The experiments were performed for three times (n = 3). All flow cytometry data analysis was performed using MoFlo XDP software and plots were generated using Flow Jo.

### RNA extraction

Medium was aspirated and ADSCs were washed twice by pre-chilled PBS buffer. PBS was removed completely before 1 mL TRI reagent was added into each well (6-well plate) and incubated for 5 min. The cell lysate in TRI reagent was then transferred into a 1.5 mL Eppendorf tube, which was shaked vigorously for about 15 s after 250 µL chloroform was added. The tube was kept still at room temperature for 2–5 min in prior to centrifuge (12,000 g, 15 min, 4 C). At this point, three layers would be observed: top, clear aqueous; middle, white precipitated DNA; bottom, pink organic phase. The top aqueous phase was carefully transferred into another 1.5 mL Eppendorf tube and 550 µL isopropanol was added. The Eppendorf tube was left at room temperature for 5 min after being inverted for several times. The mixture was centrifuged at 14,000 rpm for 20 min and the supernatant was aspirated afterwards. 1.3 mL 75% ethanal was added to resuspend the precipitate. Another centrifuge was conducted and the supernatant was discarded. The pellet (RNA) was dried in air. The RNA sample was dissolved in 50 µL DEPC treated ddH2O. The absorbance at 260 nm (A260) and the ratio of A260/A280 was measured on NanoDrop for calculating the concentration and purity of the RNA sample. The concentration was calculated according to the equation: RNA concentration (µg/µL) = A260 * 40 * 200 * 10^−3^. The sample can be stored at −80 °C freezer for further use. The experiments were performed for three times (n = 3).

### Reverse transcription of RNA

For each reaction, the following were mixed together in a RNase free tube to make a final volume of 12 µL: 0.1 ng-5 µg RNA, 1 µL oligo (dT) or miRNA specific primer, DEPC treated ddH2O. After 5-min incubation at 65 C, 4 µL 5x reaction buffer, 2 µL 10 mM dNTP, 1 µL RNAase inhibitor, and 1 µL reverse transcriptase was added and mixed gently. The reaction was kept at 37 °C for 1 h, followed by 5-min heat-inactivation at 70 °C. Reaction mixture can be stored at −80 °C.

### Quantitative Real-time PCR (qRT-PCR)

For accurate results, a 2x master mix containing polymerases, detection reagents should be used to minimized pipetting error. For each sample, triplicates were conducted and uniform amount of RNA should be used for the cDNA synthesis reaction or otherwise, a uniform amount of the cDNA reaction should be added to the qRT-PCR master mix. For each reaction, 10 µL of 2x mater mix, 1 µL of RCR forward and reverse primer (10 µM), 1 µL of cDNA reaction, 7 µL ddH_2_O was used. Reaction setup: 3 min 95 °C, 40 cycles (12 s 95 °C, 40 s 62 °C)^50^. The primer secquences were shown in Supplementary Table S2).

### qRT-PCR data analysis with double delta Ct analysis

The average of the Ct values was calculated for the genes being tested in the experimental and control conditions and also the reference genes. ∆Cts for the experimental and control conditions were then calculated (∆Ct = Ct(target) – Ct(reference)), respectively. ∆∆Ct was calculated according to equation ∆∆Ct = ∆Ct(experimental) − ∆Ct(control). Since all the calculations are in logarithm base 2, the fold change was calculated by 2^−∆∆Ct^. Statistical analysis was conducted by SPSS 21.0 software, each value was represented by mean ± sd. T test was carried out when there are only two group, and p < 0.05 was considered as significant difference.

### Cell transfection

Cell transfection was conducted in a 6-well plate with cells at 90% confluency. The experiments were performed for three times (n = 3). Each well contained 3 × 10^5^ cells in 2 mL serum-containing, antibiotic-free DMEM. Two wells were included for control and empty vector. For each transfection, 250 µL serum-free DMEM containing 4 µg agomir was mixed thoroughly with 250 µL serum-free DMEM containing 10 µL Lipofectamin2000 after a 50-min incubation at room temperature and the mixture was kept at room temperature for 20 min in prior to being added into each well in the 6-well plate. Cells were kept in an incubator at 37 °C, 5% CO_2_ for 24 hours before medium was changed with complete growth medium. 72 h later, transfection efficiency was checked by western blot.

### Western blot

The samples to be loaded were boiled at 100 °C for 5 min. Equal amounts of loading sample (100 µg) were separated using sodium dodecyl sulfate–polyacrylamide gel electrophoresis (SDS-PAGE) in TGS 1X buffer and transferred to PVDF membranes at 4 °C overnight. After blocking with TBST (TBS with 0.1% Tween 20) containing 5% fat-free milk for 2 h at room temperature, the membranes were incubated with the indicated primary antibodies (β-actin: 1:2000, PTEN: 1:1000) at 4 °C in TBST buffer containing 1% BSA overnight. The membrane was washed five times with TBST before being incubated with horseradish peroxidase (HRP)-conjugated secondary antibodies in TBST buffer containing 1% BSA for 2 h at 37 °C. After three washes with TBST, the blots were visualized and imaged with the Peirce ECL plus western blotting substrate. The results of western blot were quantified by Imag J software.

### Exosome extraction

FBS was filtered to remove any exosomes before being used for preparing complete growth medium. When ADSCs were in exponential growth phase at 70–80% confluency, medium was changed. After 24-hour incubation at 37 °C, conditioned medium (CM) was then collected for exosome extraction. The conditioned medium was centrifuged several times to remove any debris of cells: 300 g for 5 min, supernatant was collected, 1,200 g for 20 min, supernatant was collected, repeat 1,200 g centrifuge, followed by 10,000 g for 30 min, repeat 10, 000 g centrifuge. The procedure for exosome extraction was illustrated in Fig. 2.

### Transmission electron microscopy

Negative staining technique was used in TEM imaging. The 400 mesh copper grids (#1200211, Spi Supplies) coated with continuous thick carbon film (~40 nm) were glowed prior to use in order to increase the hydrophilicity. After being loaded on the grid, samples containing exosomes (10 µL exosome extraction +10 µL PBS buffer) were stained with 3.0% w/v uranyl acetate (pH 6.8) for 5 min. The grids were rinsed by ddD_2_O twice and air-dried prior to imaging.

### Tube formation assay

The Matrigel (10 mg/mL) was kept in 4 °C refrigerator overnight to allow the formation of hydrogel. On the second day, 40 µL of gel-state Matrigel was added into each well of a 24-well plate and the plate was put into a 37 °C incubator for 2 hours to allow solidification. Cell suspension containing 2 * 10^4^ cells were added into each well (medium only, ADSCs-miR-21 agomir control-exo, ADSCs-miR-21 agomir-exo) and the plate was kept in 37 °C incubator for another 24 hours in prior to imaging. The experiments were performed for three times (n = 3). Image-Pro Plus software was used for imaging analysis and tube counting.

### Statistical analysis

Quantitative data were expressed as mean ± standard deviation (SD). Statistical comparisons were made by ANOVA analysis and two-sample Student’s t-test. *P* value < 0.05 was considered statistically significant.

## Supplementary information

Exosomes from Adipose-Derived Stem Cells (ADSCs) Overexpressing miR-21 Promote Vascularization of Endothelial Cells

## Data Availability

The data that support the finding of this study are available from the corresponding author on reasonable request.
