# Supplementary material for: Exosomes from Adipose-Derived Stem Cells (ADSCs) Overexpressing miR-21 Promote Vascularization of Endothelial Cells
Source: Sci Rep. 2019 Sep 6;9:12861. doi: 10.1038/s41598-019-49339-y (PMC6731308; doi:10.1038/s41598-019-49339-y)
Supplement: Supplementary file 1 — Exosomes from Adipose-Derived Stem Cells (ADSCs) Overexpressing miR-21 Promote Vascularization of Endothelial Cells [file 41598_2019_49339_MOESM1_ESM.pdf]

Supplementary Information for

Exosomes from Adipose-Derived Stem Cells (ADSCs) Overexpressing miR-21 Promote Vascularization of Endothelial Cells

Yang An*, Jianfang Zhao, Fangfei Nie, Zelian Qin, Hongyu Xue, Guanhuier Wang, Dong Li*

Department of Plastic Surgery, Peking University Third Hospital

Medical Beauty Center, Peking University Third Hospital, No. 49 Huayuan North Road, Haidian District, Beijing, 100191

Correspondence and requests for materials should be addressed to Yang An (anyangdoctor@163.com) and Dong Li (Lidong9@sina.com).

Table of Contents

Figure S1. Flow cytometric analysis of ADSC surface markers CD29………………………...1

Figure S2. Flow cytometric analysis of ADSC surface markers CD44……………………..…..1

Figure S3. Flow cytometric analysis of ADSC surface markers CD49d ………………………1

Figure S4. Flow cytometric analysis of ADSC surface markers CD34………………………...2

Figure S5. Protein Concentration measurement in isolated isolated from ADSCs. 3

Figure S6. Melting curves of the miR-21 and U6 amplification assay..................................... 6

Table S1. Summary of the length of the tubes formed by HUVEC cells treated by ADSCs-miR-21 agomir control-exosomes, ADSCs-miR-21 agomir exosomes for 8 hours, as well as control group treated by nothing. 6

Table S2. qRT-PCR primer design……………………………………………………………..6

Figure S7. Melting curves of GAPDH, HIF-1α, VEGF, SDF-1amplification assay................ 10

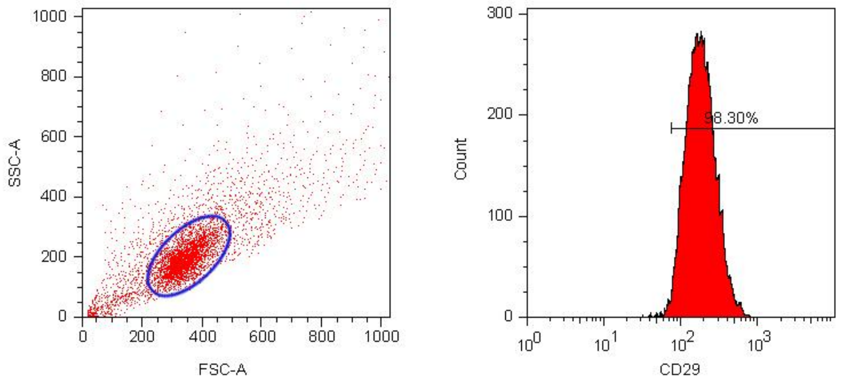

Figure S1. Flow cytometric analysis of ADSC surface markers CD29.

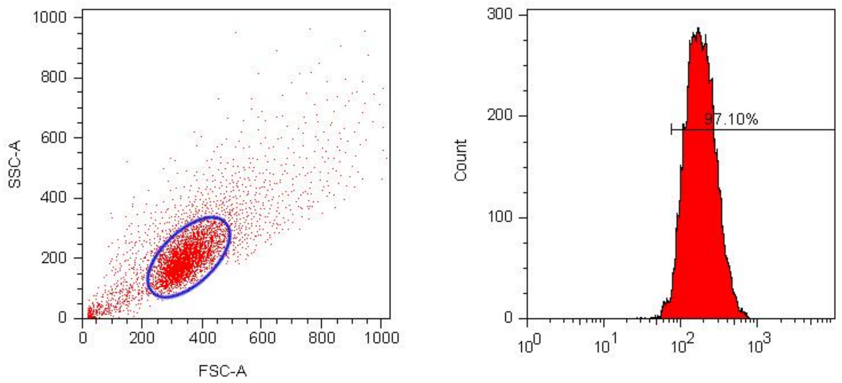

Figure S2. Flow cytometric analysis of ADSC surface markers CD44.

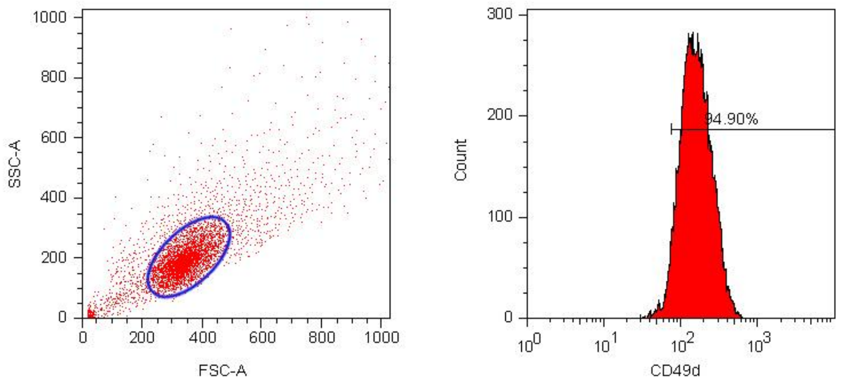

Figure S3. Flow cytometric analysis of ADSC surface markers CD49d.

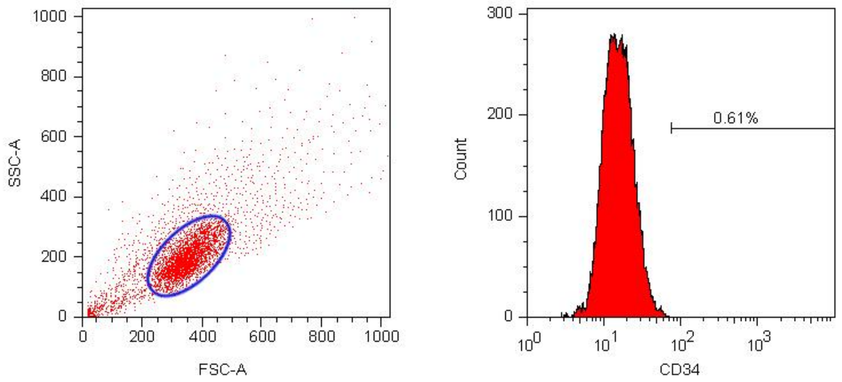

Figure S4. Flow cytometric analysis of ADSC surface markers CD34.

O.D.	Concentration (μg/mL)
0.7086	2000
0.6801	1500
0.465	1000
0.4167	750
0.3212	500
0.2199	250
0.1862	125
0.1257	25
0.1109	0

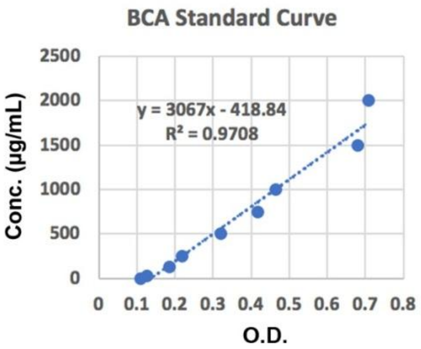

	ADSCs-miR-21 agomir control	ADSCs-miR-21 agomir
OD	0.5967	0.6083
Conc. (μg/mL)	1411.2389	1446.8161

Figure S5. Protein concentration measurement in isolated isolated from ADSCs.

U6

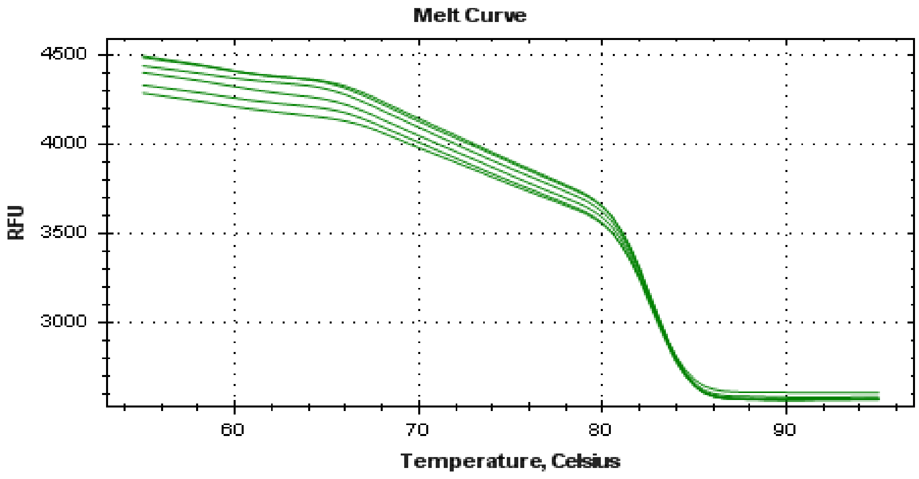

Melt Curve
4500
4000
3500
3000
RFU
60
70
80
90
Temperature, Celsius

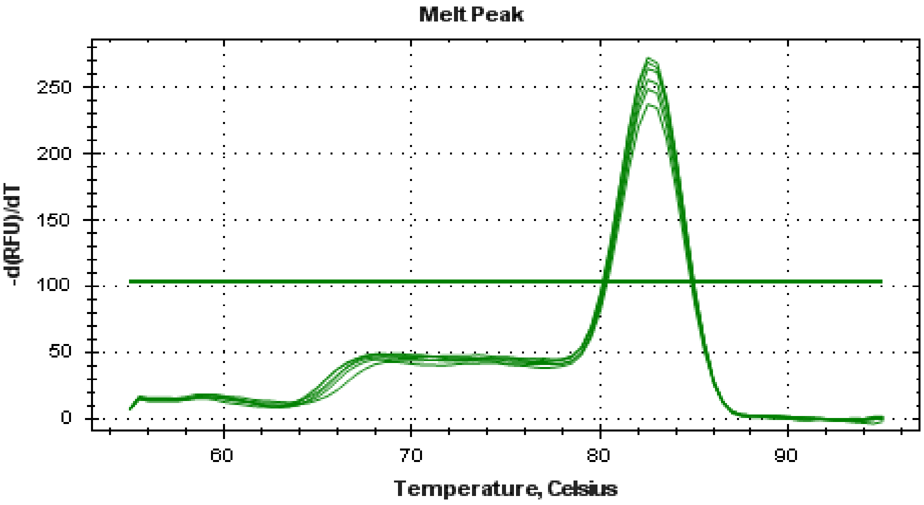

Melt Peak
250
200
150
100
50
0
-d(RFU)/dT
60
70
80
90
Temperature, Celsius

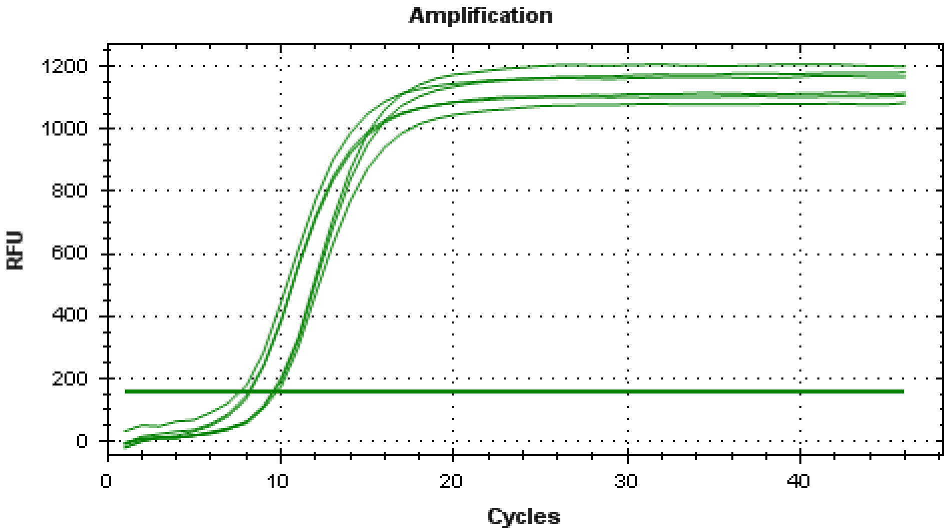

Amplification
1200
1000
800
600
400
200
0
RFU
0
10
20
30
40
Cycles

miR-21

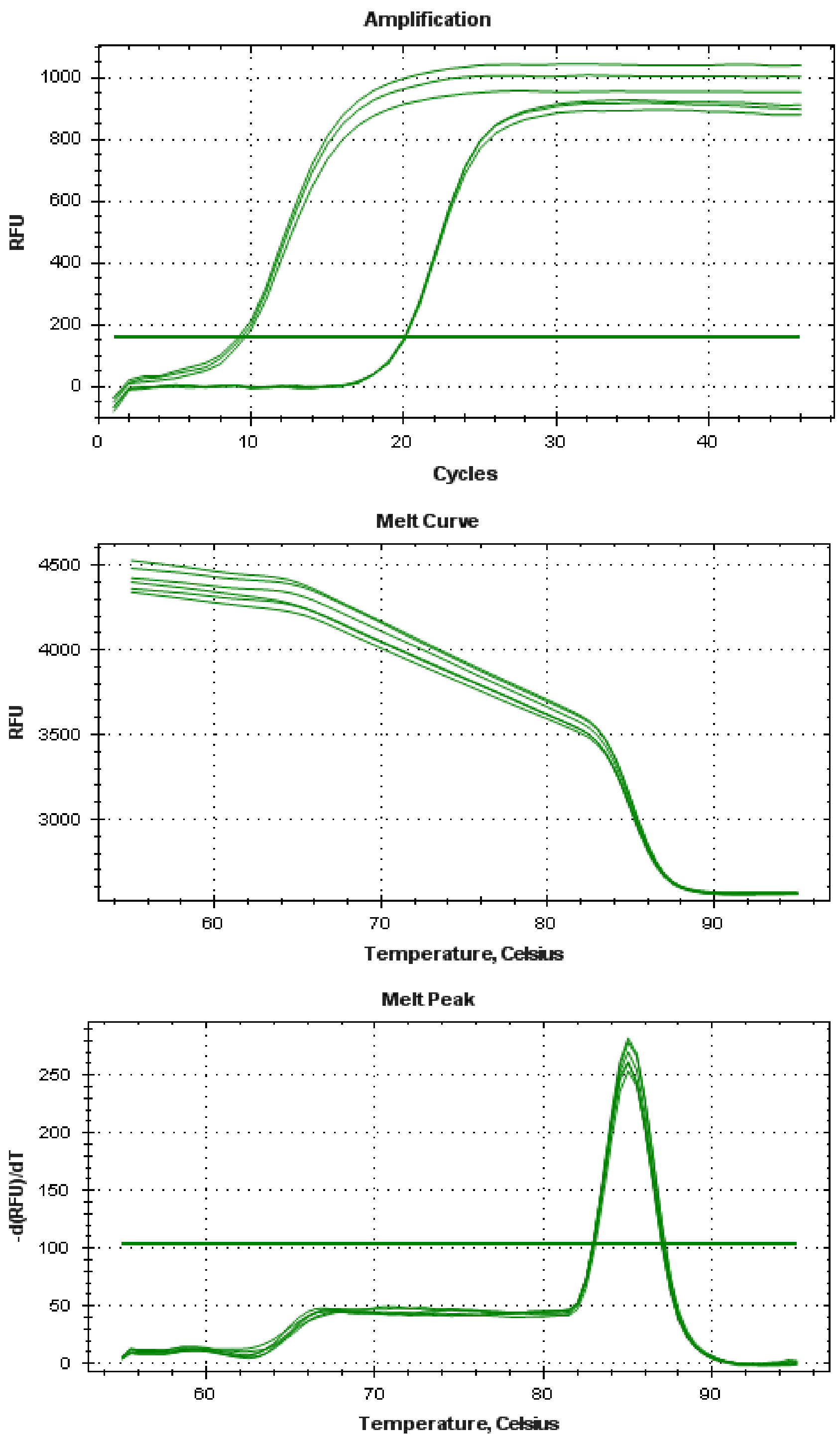

Figure S6. Melting curves of the miR-21 and U6 amplification assay.

Table S1. Summary of the length of the tubes formed by HUVEC cells treated by ADSCs-miR-21 agomir control-exosomes, ADSCs-miR-21 agomir exosomes for 8 hours, as well as control group treated by nothing.

	Nb branches	Total length	Total segments length	
**1**	30	5535	2700	control
**2**	64	7705	2835	
**3**	39	5339	2126	
**4**	50	5939	2624	Control-exosomes
**5**	31	5343	2160	
**6**	61	9069	3124	
**7**	42	6692	3917	miR-21 exosomes
**8**	41	7098	4620	
**9**	49	6686	3724	

Table S2. qRT-PCR primer design

Gene	Sense(Forward Primer)	Antisense(Reverse Primer)
U6	CTCGCTTCGGCAGCACATATACT	ACGCTTCACGAATTTGCGTGTC
miR-21-RT	GTCGTATCCAGTGCAGGGTCCGAGG TATTCGCACTGGATACGACTCAACA	
miR-21	GCGCGCTAGCTTATCAGACTGA	GTGCAGGGTCCGAGGT
HIF1-a	TGCTTGGTGCTGATTTGTGA	GGTCAGATGATCAGAGTCCA
VEGF	TTGGTGCTACTGTTTATCCG	TATGTACTACGGAATATCTCG
SDF-1	AAAAGAATTCATGAACGCCAAGGTC GTG	AAAGGTACCATCTTGAACTTGTTTAA AG
GAPDH	TGGTATGACAACGAATTTGG	TCTACATGGCAACTGTGAGG

GAPDH

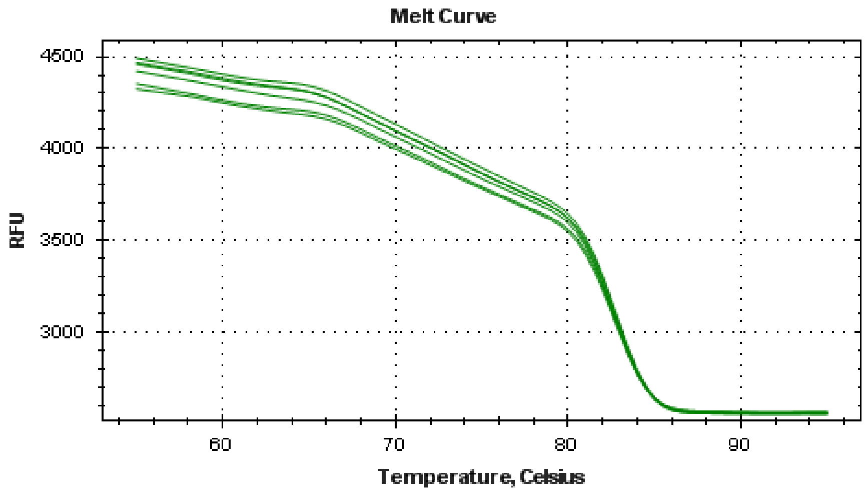
Melt Curve
4500
4000
3500
3000
RFU
60
70
80
90
Temperature, Celsius

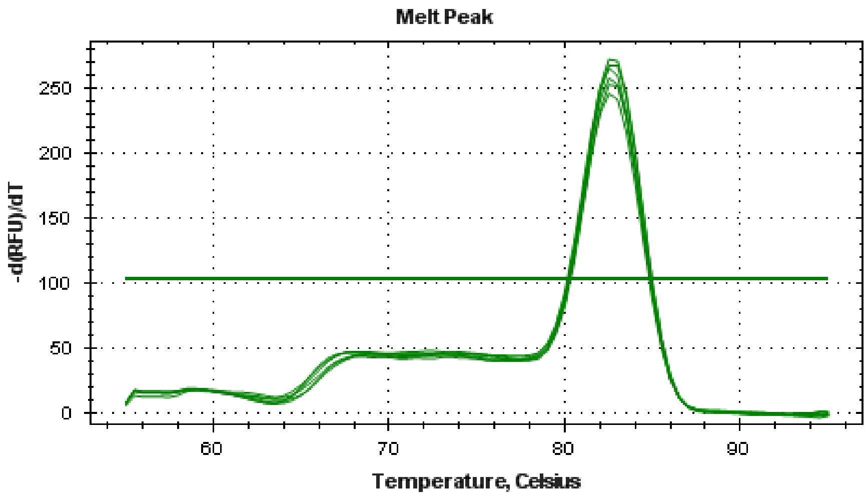
Melt Peak
250
200
150
100
50
0
-d(RFU)/dT
60
70
80
90
Temperature, Celsius

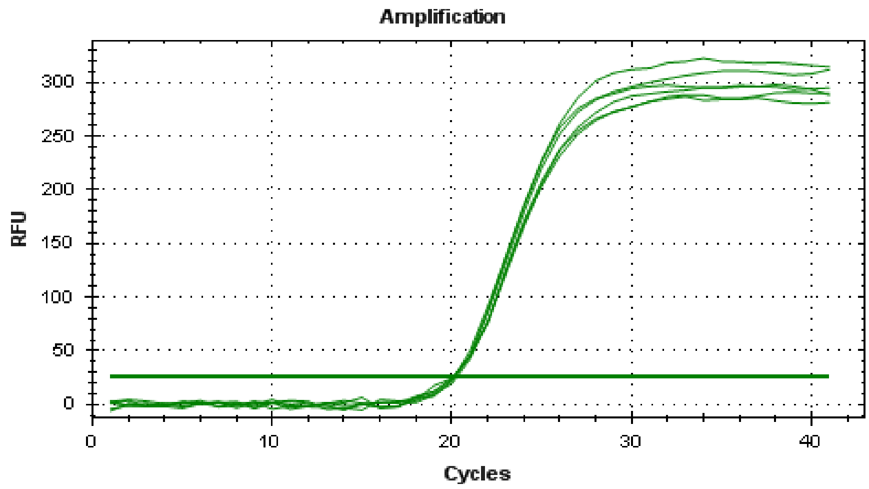
Amplification
300
250
200
150
100
50
0
RFU
0
10
20
30
40
Cycles

HIF1-α

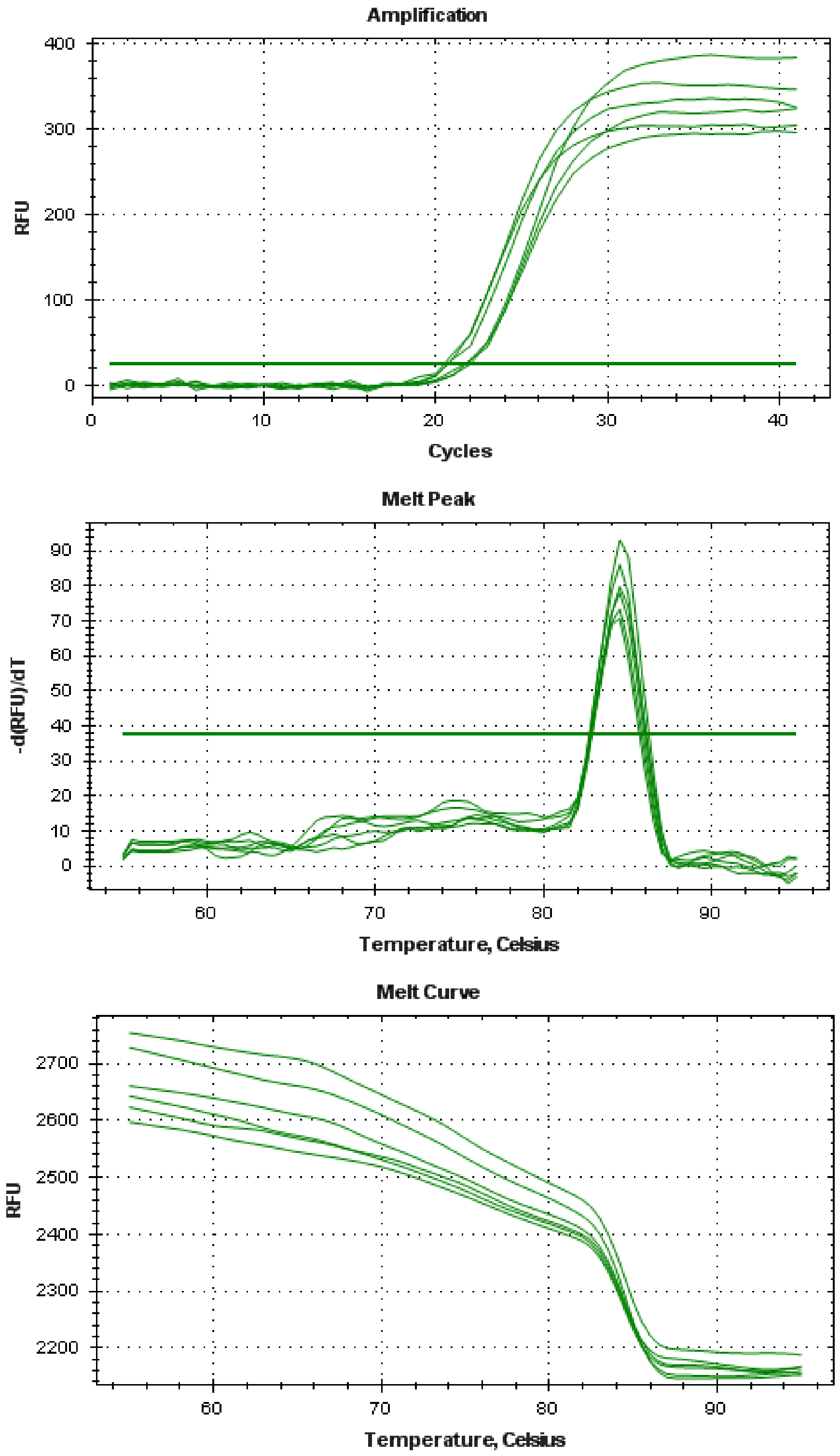

VEGF

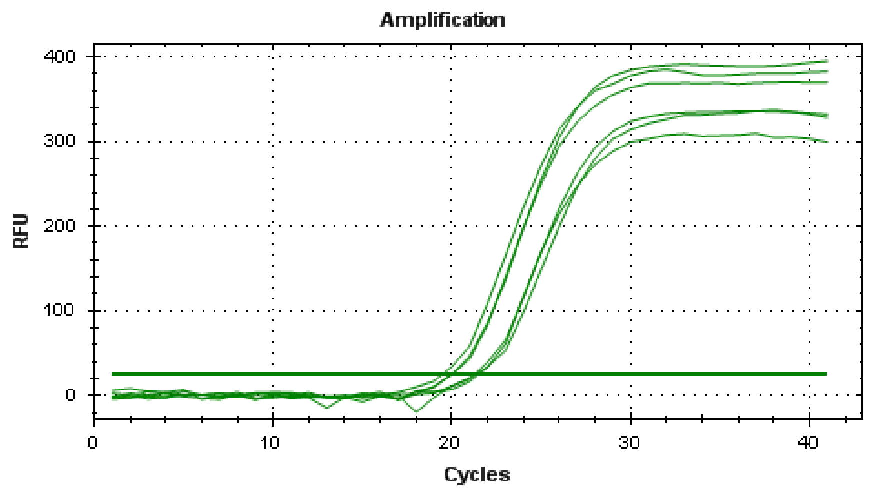
Amplification
RFU
400
300
200
100
0
0
10
20
30
40
Cycles

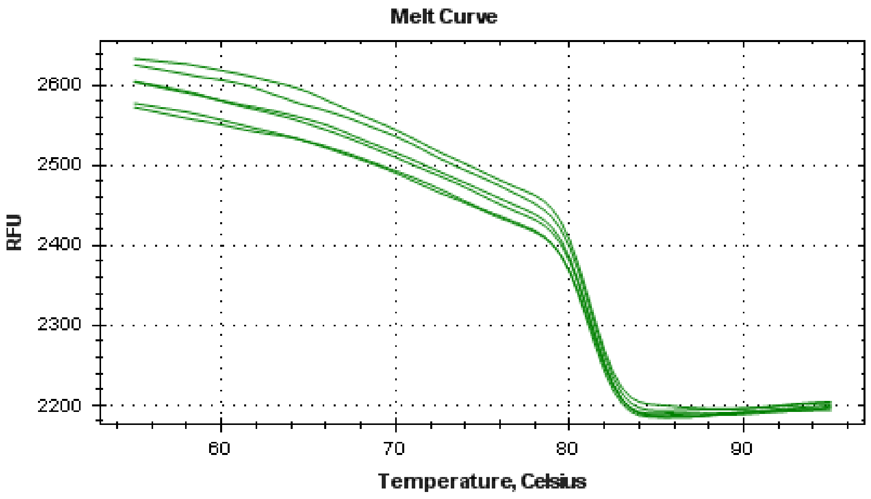
Melt Curve
RFU
2600
2500
2400
2300
2200
60
70
80
90
Temperature, Celsius

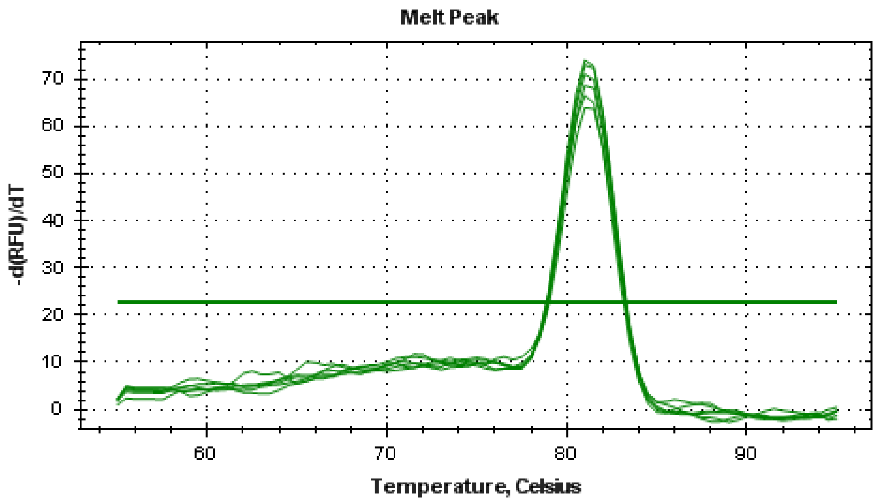
Melt Peak
-d(RFU)/dT
70
60
50
40
30
20
10
0
60
70
80
90
Temperature, Celsius

SDF-1

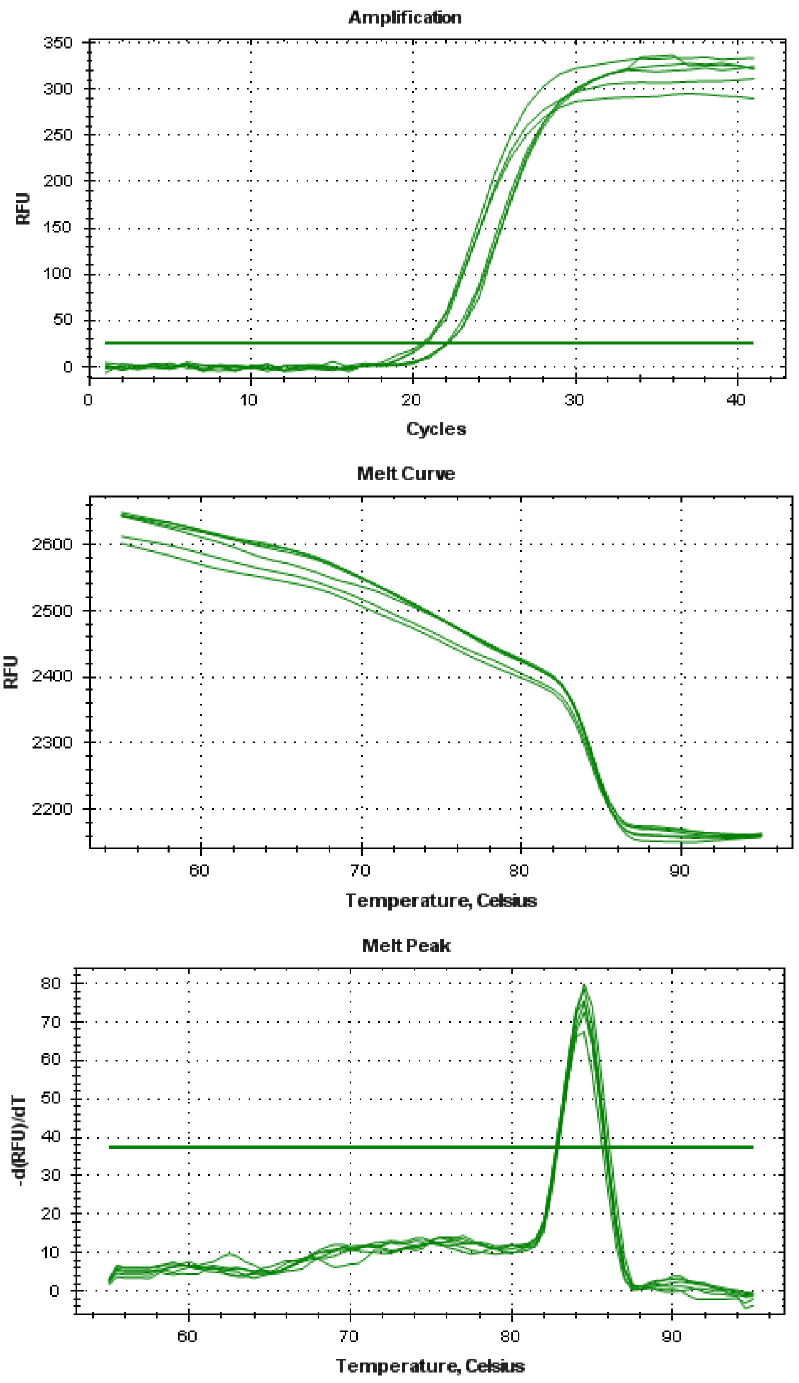

Figure S7. Melting curves of GAPDH, HIF1-α, VEGF, SDF-1 amplification assay.
